# Proliferation and Survival Signaling from Both Jak2-V617F and Lyn Involving GSK3 and mTOR/p70S6K/4EBP1 in PVTL-1 Cell Line Newly Established from Acute Myeloid Leukemia Transformed from Polycythemia Vera

**DOI:** 10.1371/journal.pone.0084746

**Published:** 2014-01-03

**Authors:** Toshikage Nagao, Tetsuya Kurosu, Yoshihiro Umezawa, Ayako Nogami, Gaku Oshikawa, Shuji Tohda, Masahide Yamamoto, Osamu Miura

**Affiliations:** 1 Department of Hematology, Graduate School of Medical and Dental Sciences, Tokyo Medical and Dental University, Tokyo, Japan; 2 Department of Laboratory Medicine, Graduate School of Medical and Dental Sciences, Tokyo Medical and Dental University, Tokyo, Japan; Wayne State University, United States of America

## Abstract

The gain of function mutation *JAK2-V617F* is very frequently found in myeloproliferative neoplasms (MPNs) and is strongly implicated in pathogenesis of these and other hematological malignancies. Here we report establishment of a new leukemia cell line, PVTL-1, homozygous for *JAK2-V617F* from a 73-year-old female patient with acute myeloid leukemia (AML) transformed from MPN. PVTL-1 is positive for CD7, CD13, CD33, CD34, CD117, HLA-DR, and MPO, and has complex karyotypic abnormalities, 44,XX,-5q,-7,-8,add(11)(p11.2),add(11)(q23),−16,+21,−22,+mar1. Sequence analysis of *JAK2* revealed only the mutated allele coding for Jak2-V617F. Proliferation of PVTL-1 was inhibited and apoptosis was induced by the pan-Jak inhibitor Jak inhibitor-1 (JakI-1) or dasatinib, which inhibits the Src family kinases as well as BCR/ABL. Consistently, the Src family kinase Lyn was constitutively activated with phosphorylation of Y396 in the activation loop, which was inhibited by dasatinib but not by JakI-1. Further analyses with JakI-1 and dasatinib indicated that Jak2-V617F phosphorylated STAT5 and SHP2 while Lyn phosphorylated SHP1, SHP2, Gab-2, c-Cbl, and CrkL to induce the SHP2/Gab2 and c-Cbl/CrkL complex formation. In addition, JakI-1 and dasatinib inactivated the mTOR/p70S6K/4EBP1 pathway and reduced the inhibitory phosphorylation of GSK3 in PVTL-1 cells, which correlated with their effects on proliferation and survival of these cells. Furthermore, inhibition of GSK3 by its inhibitor SB216763 mitigated apoptosis induced by dasatinib but not by JakI-1. Together, these data suggest that apoptosis may be suppressed in PVTL-1 cells through inactivation of GSK3 by Lyn as well as Jak2-V617F and additionally through activation of STAT5 by Jak2-V617F. It is also speculated that activation of the mTOR/p70S6K/4EBP1 pathway may mediate proliferation signaling from Jak2-V617F and Lyn. PVTL-1 cells may provide a valuable model system to elucidate the molecular mechanisms involved in evolution of Jak2-V617F-expressing MPN to AML and to develop novel therapies against this intractable condition.

## Introduction

The cytoplasmic tyrosine kinase Jak2 plays a crucial role in regulation of proliferation and apoptosis of hematopoietic cells by coupling with a variety of cytokine receptors, such as those for erythropoietin and IL-3, to activate various signaling pathways including the STAT5, Ras/Raf-1/MEK/Erk, and phosphatidylinositol 3′-kinase (PI3K)/Akt pathways [1], [2]. A somatic mutation of *JAK2*, coding for constitutively activated Jak2-V617F, is frequently observed in *BCR/ABL1*-negative myeloproliferative neoplasms (MPNs): 96% in polycythemia vera (PV), 55% in essential thrombocythemia (ET), and 65% in primary myelofibrosis (PMF) [3]. The *JAK2-V617F* mutation is also found, though much less frequently, in various other hematological malignancies, including acute myeloid leukemia (AML) and myelodysplastic syndrome (MDS), underscoring the importance of Jak2 in regulation of hematopoiesis. Jak2-V617F is constitutively activated without cytokine stimulation and stimulates the various downstream signaling pathways that are normally activated by cytokine-stimulated Jak2, such as the STAT5, MEK/Erk and PI3K/Akt pathways, thus leading to cytokine-independent cell survival and proliferation when expressed in cytokine-dependent hematopoietic cell lines [2], [4]. Moreover, various murine models have demonstrated that Jak2-V617F causes a phenotype similar to PV [5]. A number of Jak2 inhibitors have been developed and under clinical trials or approved for clinical use against MPNs with limited success, which is partly because of their inherent myelo-suppressive effects due to inhibition of normal Jak2 [4]. Although some cases of PV, and less frequently those of ET, progress and transform into MDS or AML, the significance of Jak2-V617F in the evolution of diseases remains unknown, because the progression dose not correlate with the presence or allele burden of *JAK2-V617F* [3]. Furthermore, *JAK2-V617F*-negative AML may arise from *JAK2-V617F*-positive PV or ET cases [6].

The Src family of cytoplasmic tyrosine kinases (SFKs), such as Lyn, Hck, and Fgr, also play important roles in regulation of survival and proliferation of normal or malignant hematopoietic cells downstream of the cytokine and growth factor receptors or aberrantly activated tyrosine kinase mutants, such as BCR/ABL [7]. We previously reported that Lyn was activated downstream of Epo-stimulated Jak2 and involved in tyrosine phosphorylation of STAT5 and the signal adaptor molecule CrkL [8], [9]. Moreover, activation of Lyn may play a role in acquisition of imatinib resistance of BCR/ABL-expressing leukemic cells [10], in which Jak2 was implicated in upregulation of the Lyn kinase activity [11]. It was also reported that the Lyn kinase activity was elevated in most of the AML samples and was implicated in leukemic cell proliferation possibly by activating the mTOR signaling pathway [12]. The mTOR complex 1 (mTORC1) plays critical roles in cell growth and proliferation by phosphorylating p70S6K and 4EBPs, including 4EBP1, to promote ribosome biogenesis and mRNA translation of proteins required for cell proliferation, such as c-Myc and Cyclin D1 [13]. However, the molecular mechanisms underlying activation of various signaling pathways by Lyn to stimulate proliferation and sustain survival of leukemic cells remain poorly understood.

Five AML cell lines with the Jak2-V617F mutation have previously been reported and utilized for various studies on Jak2-mediated signaling and leukemogenesis [14], [15]. In the present study, we establish and characterize a new leukemic cell line, PVTL-1, derived from a patient with AML evolving from PV. Intriguingly, proliferation and survival of PVTL-1 is dependent not only on Jak2-V617F but also on constitutively activated Lyn. We find that Jak2-V617 is mainly involved in activation of STAT5, while Lyn is responsible for tyrosine phosphorylation of various signaling molecules, such as SHP2, Gab2, CrkL, c-Cbl, and Shc, which form various signaling complexes. Furthermore, Jak2-V617F as well as Lyn is involved in activation of the mTOR pathway and inactivation of GSK3, which may play critical roles in proliferation and survival of PVTL-1 cells. Elucidation of the molecular mechanisms by which Jak2-V617F and Lyn protect PVTL-1 cells from apoptosis and stimulate proliferation may provide valuable information for pathogenesis of AML transformation from MPN and for development of novel therapeutic strategies against this intractable disease condition.

## Materials and Methods

### Ethics Statement

The study was approved by the ethical committee of Tokyo Medical and Dental University. Written informed consent was obtained from the patient in compliance with the Declaration of Helsinki.

### Case Report

A 58-year-old Japanese woman was referred to our hospital because of increases in blood cell counts in July 1995. Her peripheral blood counts were as follows: red blood cells 8.29×10^12^/L, hemoglobin 17.9 g/dL, platelets 1,680×10^9^/L, and white blood cells 18.7×10^9^/L. The bone marrow was hypercellular with a marked increase in megakaryocytes and normal myeloid and erythroid differentiation. The karyotype was 46,XX without the Philadelphia chromosome. These findings were compatible with the diagnosis of PV, and the patient was treated with busulfan followed by hydroxyurea for more than 10 years. The disease transformed to MDS in 2009, with the development of anemia, trilineage dysplasia in the bone marrow, and the complex karyotypic abnormalities in the bone marrow cells: 45,XX,der(5;17)(p10;q10) [11]/45,XX,der(5;7)(p10;q10),add(11)(p11.2) [5]/46,XX [4]. In March 2010, at the age of 73, the disease further progressed to AML with the peripheral blood findings of hemoglobin 6.3 g/dL, platelets 79×10^9^/L, and white blood cells 57.8×10^9^/l with 40% myeloblasts. The bone marrow was hypercellular with remarkable trilineage dysplasia and 40.8% of atypical myeloblasts. The leukemic cells were positive for surface CD7 (47.3%), CD13 (82.9%), CD33 (72.9%), CD34 (74.3%), CD56 (24.3%), and HLA-DR (59.9%), but negative for CD3, CD19, CD41, and glycophorin A. The karyotype was 42,XX, –5, –7, –8, add(11)(p11.2), −16, add(17)(p11.2), add(22)(q11.2) [2]/43, idem, +del(5)(q?), −add(11), +idic(11)(p11.2), −add(17), +mar [1]/45, XX, der(5;7)(p10;q10), −11, −20, add(22)(q11.2), +mar1, +mar2 [4]. The patient responded poorly to cytarabine-containing chemotherapy and died of multiple organ failure caused by the progression of disease in June 2010.

### Establishment of the PVTL-1 Cell Line

Peripheral blood was taken from the patient at the diagnosis of leukemic transformation in April 2010. Mononuclear cells were separated by Ficoll-Hypaque density gradient centrifugation and cultured in RPMI1640 medium with 10% FCS. The medium was exchanged every 3–5 days depending on the cell growth rate. After observing prolonged growth and confirming that the cells started to grow again after conventional freeze-thaw procedure, the cell line was considered to be established and designated as PVTL-1. The cells have been cultured continuously over 3 years at this time.

### Cells and Reagents

The human erythroleukemia cell line HEL was obtained from the Fujisaki Cell Center (Okayama, Japan) and cultured in RPMI1640 medium with 10% FCS. Ton.B210, a clone of murine IL-3-dependent BaF3 cells transfected with a BCR/ABL cDNA under the control of a tetracycline-inducible promoter, was kindly provided by Dr. G. Daley [16]. Ton.B210 cells were cultured in 10% FCS-containing RPMI 1640 medium supplemented either with 10% Wehi3B conditioned medium as the source of IL-3 or with 1 µg/ml doxycycline, which induces the expression of BCR/ABL. BaF3/E, a clone of BaF3 cells expressing the wild-type Epo receptor, was previously described [17] and cultured in 10% FCS-containing RPMI 1640 medium supplemented with 1 U/ml Epo. BaF3/E/Jak2-VF cells were obtained by infection of BaF3/E cells with a retroviral expression vector, pRx-Jak2-V617F, constructed by subcloning the coding region of Flag-Jak2-V617F from pMSCV-Jak2-V617F [18], kindly provided by Dr. R. Skoda, into the pRxZiN vector obtained from the Riken Gene Bank (Ibaraki, Japan), essentially as described previously [19]. Recombinant human Epo and imatinib were kindly provided by Chugai Pharmaceutical Co. Ltd. (Tokyo, Japan) and Novartis (Basel, Switzerland), respectively. Dasatinib and the pan-Jak inhibitor Jak inhibitor I (JakI-1) were purchased from Toronto Research Chemicals Inc. (Toronto, Canada) and Calbiochem (La Jolla, CA, USA), respectively. MK-2206 and GDC-0941 were purchased from Selleck (Houston, TX) and Chemdea (Ridgewood, NJ), respectively. DiOC6 and propidium iodide (PI) were purchased from Invitrogen (Carlsbad, CA, USA), and Sigma (St Louis, MO, USA), respectively.

Antibodies against Caspase-3 (CS-9662), cleaved Caspase-3 (CS-9661), phospho-Y705-STAT3 (CS-9131), phospho-Y694-STAT5 (CS-9359), phospho-Y1007/1008-Jak2 (CS-3776), phospho-T202/Y204-Erk (CS-9106), GSK3ß (CS-9315), phospho-S21/9-GSK3α/ß (CS-9331), phospho-S9-GSK3ß (CS-5558), 4EBP1 (CS-9644), non-phospho-T46-4EBP1 (CS-4923), phospho-T37/46-4EBP1 (CS-2855), mTOR (CS-2983), phospho-S2448-mTOR (CS-5536), p70S6K (CS-2708), phospho-T389-p70S6K (CS-9234), phospho-S240/244-S6RP, phospho-S422-eIF4B (CS-3591), Akt (CS-4691), phospho-T308-Akt (CS-9275), and phospho-S473-Akt (CS-9271) were purchased from Cell Signaling Technology (Beverly, MA, USA). Antibodies against Lyn (SC-15), STAT5A (SC-1081), SHP1 (SC-7289), SHP2 (SC-280), c-Cbl (SC-170), CrkL (SC-319), c-Abl (SC-131), and STAT3 (SC-7179) were from Santa Cruz Biotechnology (Santa Cruz, CA, USA). Antiphosphotyrosine (4G10, 05–321), anti-Jak2 (06–570), and anti-Gab2 (06–967) antibodies were from Millipore (Billerica, MA, USA). Anti-phospho-Y396-Lyn (Ab40600) and anti-ß-actin (A1978) antibodies were purchased from Abcam Cambridge, MA, USA) and Sigma (Oakville, ON, USA), respectively.

### Morphological, Immunophenotypic, and Cytogenetic Analyses

For morphological analysis, cells were cytocentrifuged and observed after the standard May-Giemsa staining procedures. Immunophenotypes of cells were examined by the standard flow cytometric analysis using various antibodies against cell surface antigens described (SRL Inc., Tokyo, Japan). Chromosome analyses were performed on short-term culture of the bone marrow cells or PVTL-1 cells using the standard Giemsa banding technique (SRL Inc.).

### Detection of *JAK2-V617F* Mutation

Genomic DNA was extracted from the patient’s peripheral blood white blood cells or PVTL-1 cells and analyzed by the allele-specific PCR method for the *JAK2-V617F* mutation as described previously [20]. The mutation was then confirmed by directly sequencing the 364-bp PCR product obtained for the internal PCR control in both directions.

### Analyses of Cell Proliferation, Viability, and Apoptosis

Cell proliferation and viability were assessed by counting viable and nonviable cell numbers by the trypan blue dye exclusion method. Cell viability was calculated by dividing number of viable cells by that of total cells. Viable cell numbers were also assessed by the sodium 3′-[1-(phenylaminocarbonyl)-3,4-tetrazolium]-bis (4-methoxy-6-nitro)benzene sulfonic acid hydrate (XTT) colorimetric assay using the Cell Proliferation Kit II (Roche Molecular Biochemicals, Mannheim, Germany), according to the manufacture’s instructions. For combination studies, the synergy was assessed with the combination index (CI) of Chou and Talalay method using CompuSyn software [21]. The CI values less than 0.9 indicate synergism. For analysis of cell cycle and apoptosis, cells were treated with Krishan’s reagent (0.05 mg/ml propidium iodide (PI), 0.1% Na citrate, 0.02 mg/ml ribonuclease A, 0.3% NP-40) for 30 min on ice and analyzed by flow cytometry. Apoptosis was also analyzed by flow cytometric analysis of cells stained with Annexin V-FITC and PI using the TACS Annexin V Kit (Trevigen, Gaithersburg, MD, USA).

For flow cytometric analysis of mitochondrial membrane potential, cells were stained with DiOC6 and PI to be analyzed as described previously [22]. Flow cytometric analysis of caspase-3 was performed using antibody specific for cleaved caspase-3 as described previously [22].

### Immunoprecipitation and Immunoblot Analyses

For immunoprecipitation experiments, cells were lysed in a lysis buffer containing 1% Triton X-100, 20 mM Tris-HCl (pH 7.5), 150 mM NaCl, 1 mM EDTA, 1 mM sodium orthovanadate, 1 mM phenylmethylsulfonyl fluoride and 10 µg/ml each of aprotinin and leupeptin. Cell lysates were subjected to immunoprecipitation and immunoblotting as described previously [23]. For immunoblot analysis of total cell lysates, samples were prepared by mixing an aliquot of cell lysates with an equal volume of 2X Laemmli’s sample buffer and heating at 100°C for 5 min. The results shown are representative of experiments repeated at least three times.

## Results

### Morphological, Immunophenotypic, and Cytogenetic Characterization of PVTL-1 Cells

**Figure 1:**
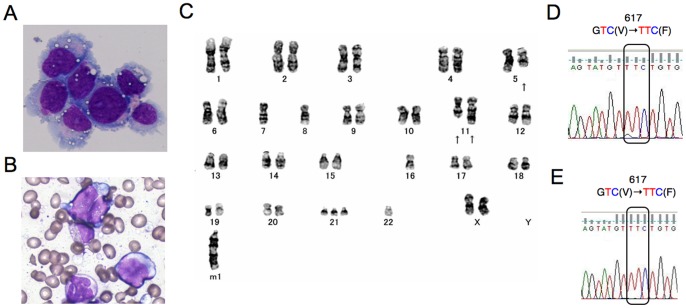
Morphological, cytogenetic, and genetic analyses of primary leukemic and PVTL-1 cells. A) A cytospin preparation of PVTL-1 cells. May-Grunwald-Giemsa staining. B) A bone marrow smear sample at leukemic transformation. C) Giemsa-banded karyotype showing 44,XX,del(5)(q?).-7,-8,add(11)(p11.2),add(11)(q23),−16,+21,−22.+mar1. Arrows indicate structurally abnormal chromosomes. D, E) Direct sequence analysis of the *JAK2* gene obtained by PCR from the peripheral blood of the patient at leukemic transformation (D) and from PVTL-1 cells (E). Nucleotide sequences around the codon coding for V617 in normal Jak2 or F617 in the Jak2 mutant are shown with the mutated codon indicated.

PVTL-1 cells showed round or oval nuclei in fine reticular pattern with several nucleoli and irregular basophilic cytoplasm with vacuoles (Fig. 1A). These features were similar to the patient’s leukemic blasts (Fig. 1B).

PVTL-1 cell were positive for surface CD7, CD13, CD33, CD34, CD117, and HLA-DR, but negative for CD3, CD19, CD41, CD56, and glycophorin A. Thus, the immunophenotypes of PVTL-1 cells were quite similar to those of the original leukemic cells except for the lack of CD56 expression, which was expressed on a subpopulation of the primary leukemic cells.

As shown in Fig. 1C, PVTL-1 cells displayed a complex abnormal karyotype, 44,XX,del(5)(q?).–7,–8,add(11)(p11.2),add(11)(q23),−16,+21,−22.+mar1, which included some chromosomal abnormalities observed commonly in primary AML cells, thus indicating that this cell line was derived from a subclone of leukemic cells evolved at the transformation to AML.

### Analysis of *JAK2-V617F* Mutation

We first analyzed the presence of *JAK2-V617F* mutation when the disease was transformed into AML in April 2010 by the allele-specific PCR method [20] and detected the 203-bp band specific for the mutation (data not shown). As shown in Fig. 1D, direct sequence analysis showed the presence of *JAK2-V617F* in 80 to 90% of the PCR product of genomic DNA obtained from the peripheral blood white blood cells, thus suggesting that the primary leukemic cells were homozygous for the *JAK2-V617F* mutation. Accordantly, only the sequences coding for Jak2-V617F were detected in PVTL-1 cells, indicating the cells are homozygous for the *JAK2-V617F* mutation (Fig. 1E).

### Dasatinib as well as Jak Inhibitor-1 Inhibited Proliferation of PVTL-1 and Induced Apoptosis

**Figure 2:**
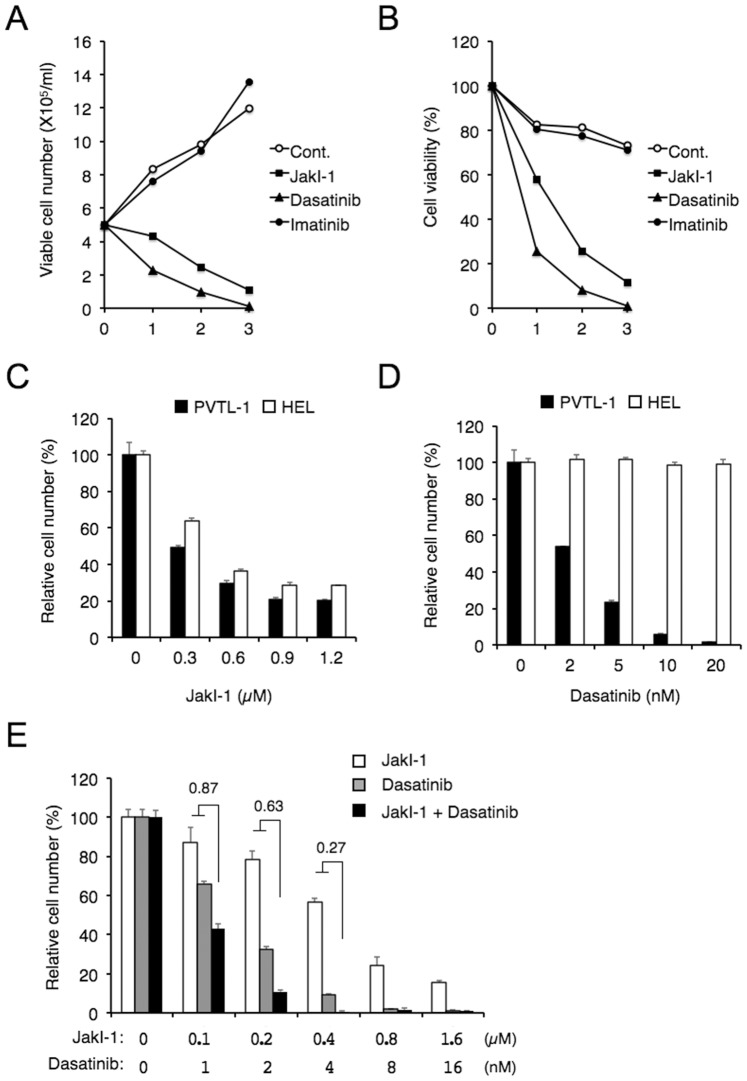
JakI-1 or dasatinib reduces proliferation and viability of PVTL-1 cells. A, B) PVTL-1 cells were plated at 5×10^5^ cells/ml in RPMI1640 medium with 10% FCS and 1 µM JakI-1, 100 nM dasatinib, or 5 µM imatinib, as indicated, for indicated days. Viable cell numbers and viability were counted and plotted in A and B, respectively. C, D) PVTL-1 or HEL cells, as indicated, were cultured with indicated concentrations of JakI-1 (C) or dasatinib (D) for 3 days and viable cell numbers were measured by XTT colorimetric assay. Each data point represents the mean of triplicate determinations, with error bars indicating standard errors, and is expressed as a percentage of cell numbers without inhibitors. E) PVTL-1 cells were cultured with indicated concentrations of JakI-1, dasatinib, or both, as indicated, for 3 days and analyzed by XTT colorimetric assay. Combination index (CI) values obtained by the method of Chou and Talalay [21] are indicated.

PVTL-1 cells proliferated in the absence of any added cytokine with the doubling time of about 2 days (Fig. 2A). To explore the significance of Jak2-V617F in PVTL-1 cells, we examined the effect of a tyrosine kinase inhibitor for the Jak family kinases, JakI-1. As shown in Fig. 2A–D, JakI-1 significantly inhibited proliferation of PVTL-1 cells in a dose-dependent manner and reduced its viability. As expected, imatinib, a first-generation tyrosine kinase inhibitor used clinically to inhibit BCR/ABL, did not show any significant effect on proliferation and viability of PVTL-1 cells (Fig. 2A, B). On the other hand, dasatinib, a second generation tyrosine kinase inhibitor for BCR/ABL, inhibited proliferation of PVTL-1 cells in a dose-dependent manner and reduced its viability more remarkably than JakI-1 (Fig. 2A, B, D). We also examined the effects of JakI-1 and dasatinib on the Jak2-V617F-expressing AML cell line HEL in comparison with PVTL-1. As shown in Fig. 2C and D, dasatinib up to 20 nM did not show any significant effect on proliferation of HEL cells, which was dose-dependently inhibited by JakI-1, in accordance with previous reports [14], [15]. It was further found that JakI-1 and dasatinib inhibited proliferation of PVTL-1 cells synergistically, as shown in Fig. 2E, with combination index values at the 25%, 50%, and 75% effective doses calculated as 0.551, 0.490, and 0.440, respectively. Because dasatinib at 2 nM, but not imatinib at 5 µM, significantly inhibited proliferation of PVTL-1 cells, it was speculated that the inhibitory effect of dasatinib on PVTL-1 cells were mediated through inhibition of SFKs, which are very efficiently inhibited by dasatinib but not significantly inhibited by imatinib [24].

**Figure 3:**
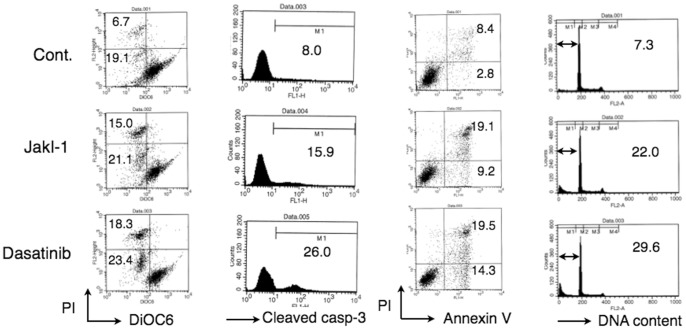
JakI-1 or dasatinib induces apoptosis involving loss of mitochondrial membrane potential and activation of caspase-3 in PVTL-1 cells. PVTL-1 cells were untreated as control (Cont.) or treated with 1 µM JakI-1 or 10 nM dasatinib, as indicated, for 48 h. Cells were then analyzed by flow cytometry for mitochondrial membrane potential with DiOC6, activation of caspase-3 with the cleavage-specific antibody, surface expression of Annexin V with anti-Annexin V, and cellular DNA content with PI, as indicated. Percentages of cells affected are indicated.

We next examined the mechanisms involved in reduction of viability of PVTL-1 cells cultured with JakI-1 or dasatinib. As shown in Fig. 3, JakI-1 or dasatinib increased the number of cells with decreased mitochondrial membrane potential and with cleaved, and thus activated, caspase-3. Moreover, these inhibitors increased the number of cells positive for Annexin V and with sub-G1 cellular DNA content (Fig. 3), which are hallmarks for apoptotic cells. Thus, these data indicate that JakI-1 and dasatinib similarly induced apoptosis in PVTL-1 cells, most likely through activation of the mitochondria-mediated intrinsic apoptotic pathway leading to activation of caspases by inhibition of Jak2-V617F and SFKs, respectively.

### Tyrosine Phosphorylation of Signaling Molecules by Jak2-V617F and Lyn in PVTL-1 Cells

**Figure 4:**
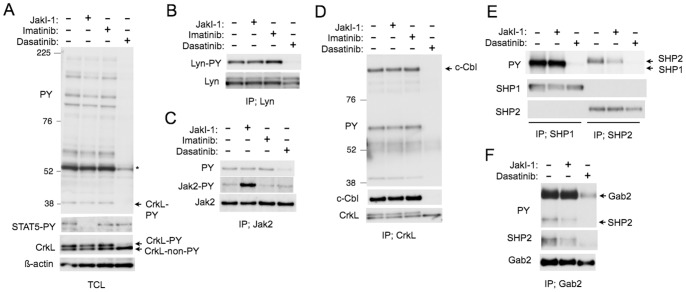
Tyrosine phosphorylation of cellular substrates and formation of signaling complexes by Jak2-V617F and Lyn in PVTL-1 cells. A–D) PVTL-1 cells were left untreated as control or treated with 1 µM JakI-1, 5 µM imatinib, or 100 nM dasatinib, as indicated for 6 h and lysed. Total cell lysates (A) and immunoprecipitates obtained with anti-Lyn (B), anti-Jak2 (C), or anti-CrkL (D) were subjected to Western blot analyses with antibodies against indicated proteins. Abbreviations used are: PY, phosphotyrosine; STAT5-PY, phospho-Y694-STAT5; Lyn-PY, phospho-Y396-Lyn; Jak2-PY, phospho-Y1007/1008-Jak2; TCL, total cell lysate; IP, immunoprecipitation. An asterisk indicates the position of the most significantly tyrosine-phosphorylated protein. Positions of tyrosine-phosphorylated and non-phosphorylated CrkL, CrkL-PY and CrkL-non-PY, respectively, are indicated. Positions of molecular weight markers are also indicated. E, F) PVTL-1 cells were left untreated as control or treated with 1 µM JakI-1or 100 nM dasatinib, as indicated for 6 h. Cell lysates were subjected to immunoprecipitation with anti-SHP1, anti-SHP2 (E), and anti-Gab2 (F) followed by Western blot analyses. Positions of relevant proteins are indicated.

We next examined the intracellular signaling mechanisms involved in regulation of proliferation and survival of PVTL-1 cells by Jak2-V617F and SFKs. First, we treated PVTL-1 cells with JakI-1, imatinib, or dasatinib and examined tyrosine phosphorylation of various signaling molecules. As shown in Fig. 4A, dasatinib and, to a much lesser degree, JakI-1, induced dephosphorylation of various cellular proteins on tyrosine. Importantly, activation-specific phosphorylation of STAT5A on Y694 was specifically inhibited by JakI-1 but not by dasatinib. Similarly, activation-specific phosphorylation of STAT3 on Y705 was also specifically inhibited by JakI-1 but not by dasatinib (Fig. S1A). On the other hand, dasatinib specifically inhibited the mobility-shift of CrkL induced by its tyrosine phosphorylation (Fig. 4A).

Because the most prominently tyrosine-phosphorylated protein corresponded in size with Lyn and was dephosphorylated by dasatinib (Fig. 4A), we next immunoprecipitated Lyn and examined its activation-specific phosphorylation on Y396 in the activation loop. As shown in Fig. 4B, the activation-specific phosphorylation of Lyn was constitutively observed and was abrogated by dasatinib. We also examined Jak2-V617F and found that it is also constitutively phosphorylated on Y1007/Y1008 in the activation loop (Fig. 4C). JakI-1 augmented tyrosine phosphorylation of Y1007/Y1008, as expected [4]. Unexpectedly, dasatinib inhibited phosphorylation of Jak2-V617 on tyrosines but not that on Y1007/Y1008 (Fig. 4C).

We next immunoprecipitated various intracellular signaling molecules and examined their tyrosine phosphorylation in PVTL-1 cells. First, CrkL was confirmed to be constitutively tyrosine phosphorylated and was found to bind c-Cbl (Fig. 4D). Dasatinib abrogated both its phosphorylation and binding with c-Cbl. The tyrosine phosphatases SHP1 and SHP2 were also constitutively phosphorylated on tyrosine, which was abrogated by dasatinib (Fig. 4E). JakI-1 partly inhibited tyrosine phosphorylation of SHP2 but not SHP1. The adaptor protein Gab2 was also constitutively phosphorylated on tyrosine and physically associated with SHP2, which was strongly inhibited by dasatinib (Fig. 4F). JakI-1 did not show any significant effect on phosphorylation of Gab2 but partly inhibited its binding with SHP2. These data suggest that Lyn is constitutively activated in PVTL-1 cells and activates signaling pathways involving the SHP2/Gab2 and CrkL/c-Cbl complexes as well as SHP1 among others, while Jak2-V617F uniquely activates STAT5 and is partly involved in phosphorylation of SHP2.

### Effects of Jak2-V617F and Lyn on Intracellular Signaling Events Regulating Proliferation and Apoptosis in PVTL-1 and HEL Cells

**Figure 5:**
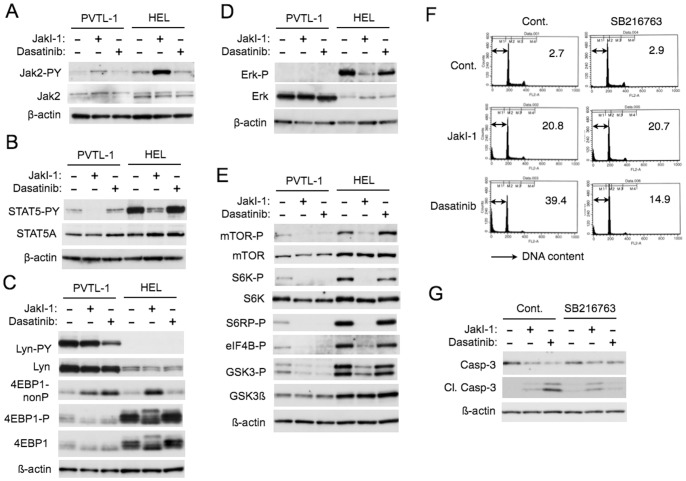
Jak2-V617F- and Lyn-mediated signaling events in PVTL-1 and HEL cells. A–E) PVTL-1 or HEL cells were treated with 1.5 µM JakI-1 or dasatinib at 20 nM (A, C, E) or 15 nM (B, D), as indicated, for 6 h (A, C, E) or 1 h (B, D) and lysed. Total cell lysates were subjected to Western blot analyses using indicated antibodies. Abbreviations used are: Jak2-PY, phospho-Y1007/1008-Jak2; STAT5-PY, phospho-Y694-STAT5; Lyn-PY, phospho-Y396-Lyn; 4EBP1-nonP, non-phospho-T46-4EBP1; 4EBP1-P, phospho-T37/46-4EBP1; Erk-P, phospho-T202/Y204-Erk; mTOR-P, phospho-S2448-mTOR; S6K-P, phospho-T389-p70S6K; S6RP-P, phospho-S240/244-S6RP; eIF4B-P, phospho-S422-eIF4B, GSK3-P, phospho-S21/9-GSK3α/ß. F) PVTL-1 cells were precultured with or without 5 µM SB216763 for 1 h and further cultured with or without 1.5 µM JakI-1 or 20 nM dasatinib, as indicated, for 24 h. Cells were analyzed for cellular DNA content with PI by flow cytometry. Percentages of apoptotic cells with sub-G1 DNA content are indicated. G) PVTL-1 cells were precultured with or without 10 µM SB216763 for 1 h and further cultured with or without 1.5 µM JakI-1 or 20 nM dasatinib, as indicated, for 24 h. Cells were lysed and subjected to Western blot analyses using indicated antibodies. Cl. Casp-3: Cleaved Caspase-3.

To gain further insights into the signaling mechanisms Jak2-V617F and Lyn regulate proliferation and survival of PVTL-1 cells, we analyzed expression and activation of these kinases in PVTL-1 cells and HEL cells comparably. As shown in Fig. 5A, activation-specific phosphorylation of Jak2-V617F on Y1007/Y1008 was observed at a significantly lower level in PVTL-1 cells than in HEL cells, which was increased by JakI-1 in both cell lines. Activation-specific phosphorylation of STAT5A on Y694, one of the major substrates of Jak2, was also observed at a much lower level in PVTL-1 cells than in HEL cells (Fig. 5B). On the other hand, Lyn was expressed at a significantly higher level in PVTL-1 cells as compared with HEL cells (Fig. 5C). Importantly, activation-specific phosphorylation of Lyn on Y397 was observed conspicuously in PVTL-1 cells, but not in HEL cells, and was downregulated by dasatinib. As shown in Fig. S1B, Jak2-V617 also failed to induce activation of Lyn in murine hematopoietic BaF3 cells expressing the Epo receptor, while BCR/ABL activated Lyn in BaF3 cells, in accordance with our previous report [25]. It was also confirmed that Lyn was expressed at a much higher level in PVTL-1 cells as compared in BaF3 cells. These observations raise a possibility that overexpression of Lyn in PVTL-1 cells may be responsible for its constitutive activation and may compensate for the relatively weak activation of the Jak2/STAT5 pathway.

Then we examined activation of various signaling events possibly downstream of Jak2-V617F and Lyn in PVTL-1 and HEL cells in comparable manners. We could detect the activated form of Erk, which was inhibited by JakI-1, in HEL cells but not in PVTL-1 cells, although the expression level of Erk in PVTL-1 cells was higher than that in HEL cells (Fig. 5D). Whereas Akt was expressed at comparable levels in PVTL-1 and HEL cells, we could not detect the activation-specific Akt phosphorylated on T308 or S473 using specific antibodies in Western blot analyses (negative data not shown). Intriguingly, however, Western blot analyses revealed that the mTOR/p70S6K/4EBP-1 pathway, known to be activated downstream of the PI3K/Akt and MEK/Erk pathways [13], was constitutively activated in PVTL-1 and HEL cells, because mTOR and its downstream effector kinase p70S6K were phosphorylated on activation-specific sites and their substrates, 4EBP-1, S6RP, and eIF4B, were constitutively phosphorylated (Fig. 5C and E). Moreover, activation of the mTOR/p70S6K/4EBP-1 pathway was significantly inhibited by JakI-1 as well as dasatinib in PVTL-1 cells and selectively by JakI-1 in HEL cells. Similarly, the inhibitory phosphorylation of GSK3α/ß on S21/9, known to be mediated mainly by Akt, was observed in these cell lines and was significantly inhibited by JakI-1 as well as dasatinib in PVTL-1 cells and selectively by JakI-1 in HEL cells (Fig. 5E). Thus, both Jak2-V617F and Lyn may be required for activation of the mTOR/p70S6K/4EBP-1 pathway as well as inhibition of GSK3α/ß in PVTL-1 cells, while Jak2-V617F alone may be involved in these signaling events in HEL cells. It is also important to note that inhibition of the mTOR/p70S6K/4EBP-1 pathway and activation of GSK3α/ß correlated with downregulation of proliferation and survival in these cells.

Although we could not detect activation-specific phosphorylation of Akt in PVTL-1 cells, we still examined the possibility that PI3K and Akt may be involved in the inhibitory phosphorylation of GSK3α/ß in PVTL-1 cells by using their specific inhibitors GDC-0941 and MK-2206, respectively. As shown in Fig. S1C, GDC-0941 inhibited the phosphorylation of GSK3α/ß in PVTL-1 cells, while MK-2206 failed to show any significant inhibitory effect. Thus, the inhibitory phosphorylation of GSK3α/ß may be regulated by PI3K in mostly Akt-independent manners in these cells. It was also confirmed that, like in PVTL-1 and HEL cells, Jak2-V617F induced the inhibitory phosphorylation of GSK3ß in BaF3 cells expressing the Epo receptor in a manner dependent on its kinase activity similarly with BCR/ABL (Fig. S1B). Because GSK3 has been strongly implicated in regulation of apoptosis [26], we examined the possible involvement of GSK3 in induction of apoptosis by JakI-1 or dasatinib in PVTL-1 cells. As shown in Fig. 5F, inhibition of GSK3 by its specific inhibitor SB216763 unambiguously mitigated apoptosis induced by dasatinib but not JakI-1. In accordance with this, SB216763 remarkably inhibited the cleavage of caspase-3 in PVTL-1 cells treated with dasatinib without showing any significant inhibitory effect on that in cells treated with JakI-1 (Fig. 5G). These results suggest that mechanisms underlying induction of apoptosis by dasatinib may mainly involve GSK3 activation, while JakI-1 may induce apoptosis also by specifically affecting other signaling pathways, such as the STAT5 pathway.

## Discussion

In the present study, we have newly established a leukemic cell line, PVTL-1, homozygous for the *JAK2-V617F* mutation from a patient with AML transformed from PV. Previously, 5 AML cell lines carrying the *JAK2-V617F* mutation have been reported: HEL, MB-02, MUTZ-8, SET-2, and UKE-1 [14], [15]. Three of them were derived from AML transformed from *BCR/ABL1*-negative MPNs: SET-2 and UKE-1 from ET and MB-02 from PMF. Except for SET-2 cells, all of these cell lines are homozygous for the Jak2-V617F mutation [15]. The homozygosity is also frequently observed in patients with PV most likely due to mitotic recombination causing loss of heterozygosity at chromosome 9p affecting *JAK2* [14], [18]. HEL and SET-2 additionally show amplification of the *JAK2* locus [15]. Intriguingly, all these cell lines exhibited losses of long-arm material from both chromosomes 5 and 7, except for UKE-1 showing monosomy 7 alone [15]. In accordance with this, PVTL-1 also showed 5q- and -7. Thus, losses of 5q and 7q may cooperate with Jak2-V617F for transformation into AML or for establishment as cell line, which needs to be explored in future studies. Also in accordance with the cell lines previously reported, PVTL-1 cells were highly sensitive to JakI-1, which reduced proliferation of PVTL-1 cells and induced apoptosis (Fig. 2 and 3). Therefore, PVTL-1 cells are dependent on Jak2-V617F and should provide a model system suitable for studies aiming to elucidate molecular mechanisms underlying progression and transformation of MPNs to AML/MDS involving Jak2-V617F.

The present study has shown that PVTL-1 cells are also dependent on or addicted to the constitutively activated Lyn kinase activity. First, dasatinib inhibited proliferation and induced apoptosis of PVTL-1 cells in a dose-dependent manner, whereas imatinib at 5 µM did not show any significant effect on these cells. Although dasatinib also inhibits c-Kit (CD117), which is expressed on PVTL-1 cells, and the PDGF receptor ß, these kinases should be also inhibited by imatinib at 5 µM [24]. These results indicate that the SFKs should play a critical role in proliferation and survival of PVTL-1 cells. Second, Lyn was constitutively phosphorylated on the activation-specific Y396 in the activation loop and comigrated with the major phosphotyrosyl protein in PVTL-1 cells, which was dephosphorylated by dasatinib (Fig. 4A, B). Although the possible involvement of other members of SFKs could not be ruled out, these results indicate that the Lyn activity should be largely responsible for proliferation and survival of PVTL-1 cells. A previous study implicated Jak2 activation by BCR/ABL in activation of Lyn in imatinib-resistant CML cells [11]. However, inhibition of Jak2-V617F by JakI-1 did not show any effect on activation-specific tyrosine phosphorylation of Lyn in PVTL-1 cells (Fig. 4B, 5C). Moreover, Jak2-V617F did not activate Lyn in HEL cells or in the BaF3 cells engineered to express Jak2-V617F (Fig. 5C and S1B), which indicates that Jak2-V617F *per se* should not cause activation of Lyn in these cells. On the other hand, because the expression level of Lyn was much higher than that in HEL or BaF3 cells (Fig. 5C, Fig. S1B), its overexpression should be, at least partly, responsible for the increased activity. Intriguingly, the Lyn overexpression has also been reported in primary CML cells with imatinib resistance and in primary CLL cells and implicated in proliferation and survival of these cells through the increased kinase activity [10], [27]. Cytogenetically, PVTL-1 cells do not show any structural abnormality of chromosome 8, which harbors the *LYN* locus at 8q12.1, but rather show monosomy 8. Nevertheless, future studies are needed to examine the possible amplification of this locus as well as the aberrant transcriptional activation of *LYN* through various mechanisms, including mutations in the regulatory region and epigenetic deregulation. It is tempting to speculate that the overexpression and activation of Lyn might have played a role in transformation of PV into AML in the present case. It should be also noted that dasatinib alone or in combination with clinically relevant Jak2 inhibitors, such as ruxolitinib, may provide an effective therapeutic strategy to treat similar cases, because dasatinib showed a more remarkable inhibitory effect on proliferation of PVTL-1 cells than JakI-1 and acted synergistically with JakI-1.

Studies using JakI-1 or dasatinib indicated that Jak2-V617F was involved uniquely in induction of tyrosine phosphorylation of STAT5, STAT3, and, at least partly, SHP2, while Lyn was involved in that of various signaling molecules, including SHP1, SHP2, CrkL, c-Cbl, and Gab2 as well as in induction of complex formation of Gab2 with SHP2 and c-Cbl with CrkL in PVTL-1 cells (Fig. 4). We previously showed that Lyn was involved in tyrosine phosphorylation of STAT5 as well as CrkL in signaling from the Epo receptor [8], [9]. Other studies have also implicated Lyn in tyrosine phosphorylation of STAT5 in several signaling pathways, including those involved in pathogenesis of AML and MPNs [28], [29]. In contrast to these observations, Jak2-V617F alone is responsible for the activation-specific tyrosine phosphorylation of STAT5 in PVTL-1 cells, while Lyn is responsible for tyrosine phosphorylation of various signaling molecules known to be involved in activation of the PI3K/Akt and MEK/Erk signaling pathways. Intriguingly, dasatinib reduced phosphorylation of Jak2-V617F on tyrosines other than Y1007/Y1008 (Fig. 4C). It has been reported that Jak2 is phosphorylated on several tyrosine residues, which regulates the kinase activity of Jak2 in either positive or negative ways [4]. Thus, although dasatinib failed to affect activation-specific phosphorylation of STAT5, a well established substrate of Jak2, it is possible that Lyn may modulate the activity of Jak2-V617F in PVTL-1 cells, which needs to be addressed in future studies.

The mTOR/p70S6K/4EBP-1 pathway, known to be activated downstream of the PI3K/Akt and MEK/Erk pathways [13], was constitutively activated in PVTL-1 as well as in HEL cells, although we failed to detect activation of Akt or Erk using activation-specific antibodies in immunoblot analyses (Fig. 5D and data not shown). Activation of this pathway was inhibited by JakI-1 as well as dasatinib in PVTL-1 cells and selectively by JakI-1 in HEL cells, thus showing correlation with proliferation and survival of these cells. Intriguingly, previous reports showed that mTOR was activated more frequently than Akt in primary AML cells most likely in a PI3K-independent manner and possibly through Lyn activation [12], [13], [30]. Furthermore, mTOR is also activated in Jak2-V617F-expressing cells, including MPN cells, and its inhibition showed a cytostatic rather than an apoptotic effect [31]. Thus, it is possible that Jak2-V617F and Lyn may stimulate proliferation of PVTL-1 cells through activation of mTOR/p70S6K/4EBP-1 pathway.

**Figure 6:**
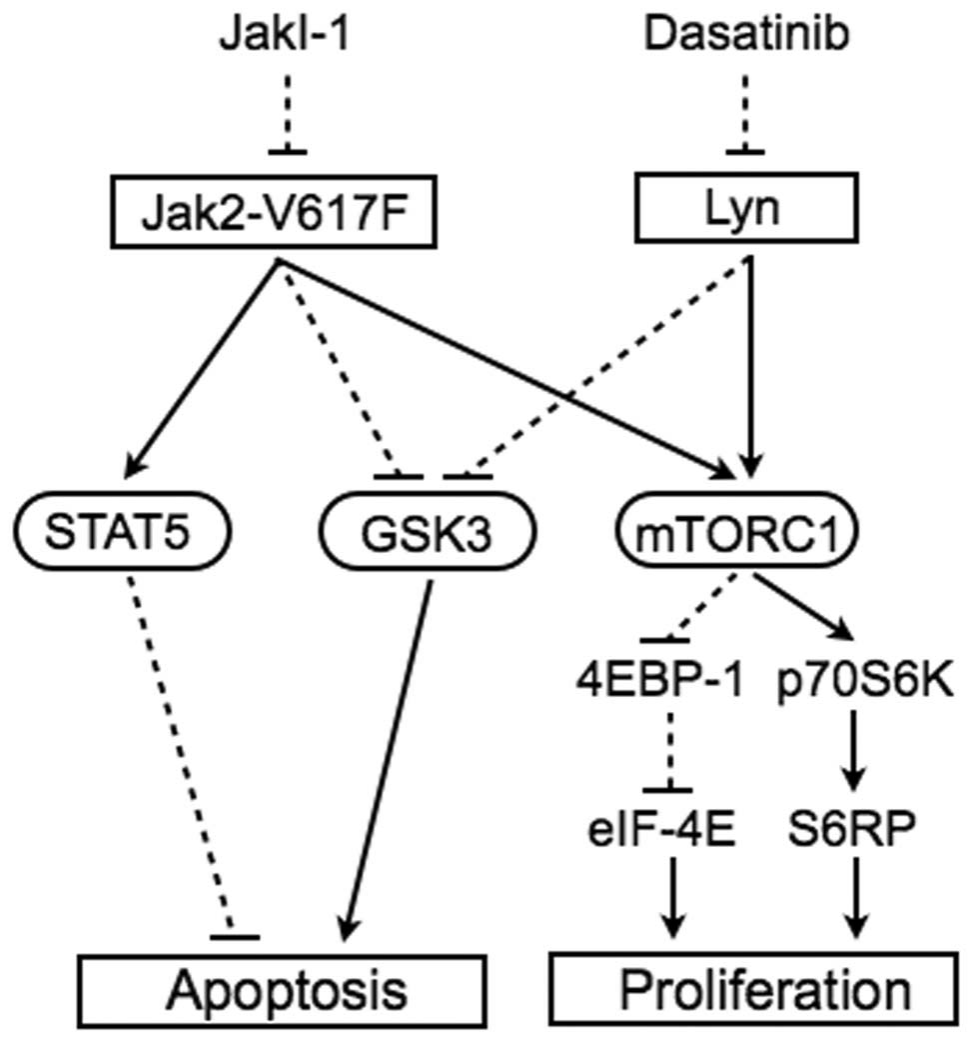
A hypothetical model of intracellular signaling mechanisms downstream of Jak2-V617F and Lyn regulating survival and proliferation of PVTL-1 cells. Arrows and dotted lines indicate stimulatory and inhibitory effects, respectively.

It is also important to note that JakI-1 and dasatinib reduced the inhibitory phosphorylation of GSK3α/ß on S21/9 (Fig. 5E), which should increase the kinase activity of GSK3 playing important roles in regulation of a variety of cellular processes, including apoptosis [26]. In this regard, we previously found that inhibition of Jak2-V617F activates GSK3 to induce degradation of Jak2-V617F and apoptosis synergistically with DNA damage-inducing chemotherapeutics [19]. Furthermore, inhibition of GSK3 activation by SB216763 very efficiently reduced apoptosis of PVTL-1 cells treated with dasatinib but not with JakI-1 (Fig. 5F, G). This difference may be explained by the fact that JakI-1 but not dasatinib additionally inhibits STAT5 activation in these cells (Fig. 4A, Fig. 5B). Based on these data, we put forward a hypothetic model (Fig. 6), in which both Jak2-V617F and Lyn inactivate GSK3 to prevent apoptosis and activate the mTOR/p70S6K/4EBP-1 pathway to promote proliferation of PVTL-1 cells, while Jak2-V617F additionally activates STAT5 to prevent apoptosis. Further studies are in progress to elucidate the mechanisms involved in regulation of GSK3 and the mTOR/p70S6K/4EBP-1 pathway by Jak2-V617F and Lyn as well as the mechanisms involved in regulation of apoptosis and proliferation downstream of these events and STAT5 activation in PVTL-1 cells. Preliminary studies using specific inhibitors suggested that the inhibitory phosphorylation of GSK3α/ß may be regulated by PI3K in mostly Akt-independent manners in these cells (Fig. S1C). Elucidation of these mechanisms would shed more light on pathogenesis of MPNs and its progression and may contribute to the development of novel therapeutic strategy against these intractable diseases.

## Supporting Information

Figure S1 **Effects of various inhibitors on intracellular signaling events in PVTL-1 and BaF3 cells expressing Jak2-V617F or BCR/ABL (A)** PVTL-1 cells were left untreated as control or treated with 1 µM JakI-1or 50 nM dasatinib, as indicated, for 6 h and lysed. Total cell lysates were subjected to Western blot analyses with antibodies against indicated proteins. STAT3-PY: phospho-Y705-STAT3. **(B)** PVTL-1 cells treated as described above, BaF3/E cells starved from Epo for 6 h, BaF3/E/Jak2-VF cells treated with or without 2 µM JakI-1 for 1 h, and BCR/ABL-expressing Ton.B210 cells teated with or wihout 3 µM imatinib for 6 h, as indicated, were subjected to Western blot analyses with antibodies against indicated proteins. Abbreviations used are: Jak2-PY, phospho-Y1007/1008-Jak2; Lyn-PY, phospho-Y396-Lyn; GSK3ß-P, phospho-S9-GSK3ß. An asterisk indicates the position of BCR/ABL. **(C)** PVTL-1 cells were left untreated as control or treated with 5 µM GDC-0941 or 5 µM MK-2206, as indicated, for 6 h and subjected to Western blot analyses. GSK3-P: phospho-S21/9-GSK3α/ß.
(TIF)
