# Supplementary figures and images for: Proliferation and Survival Signaling from Both Jak2-V617F and Lyn Involving GSK3 and mTOR/p70S6K/4EBP1 in PVTL-1 Cell Line Newly Established from Acute Myeloid Leukemia Transformed from Polycythemia Vera
Source: PLoS One. 2014 Jan 3;9(1):e84746. doi: 10.1371/journal.pone.0084746 (PMC3880321; doi:10.1371/journal.pone.0084746)

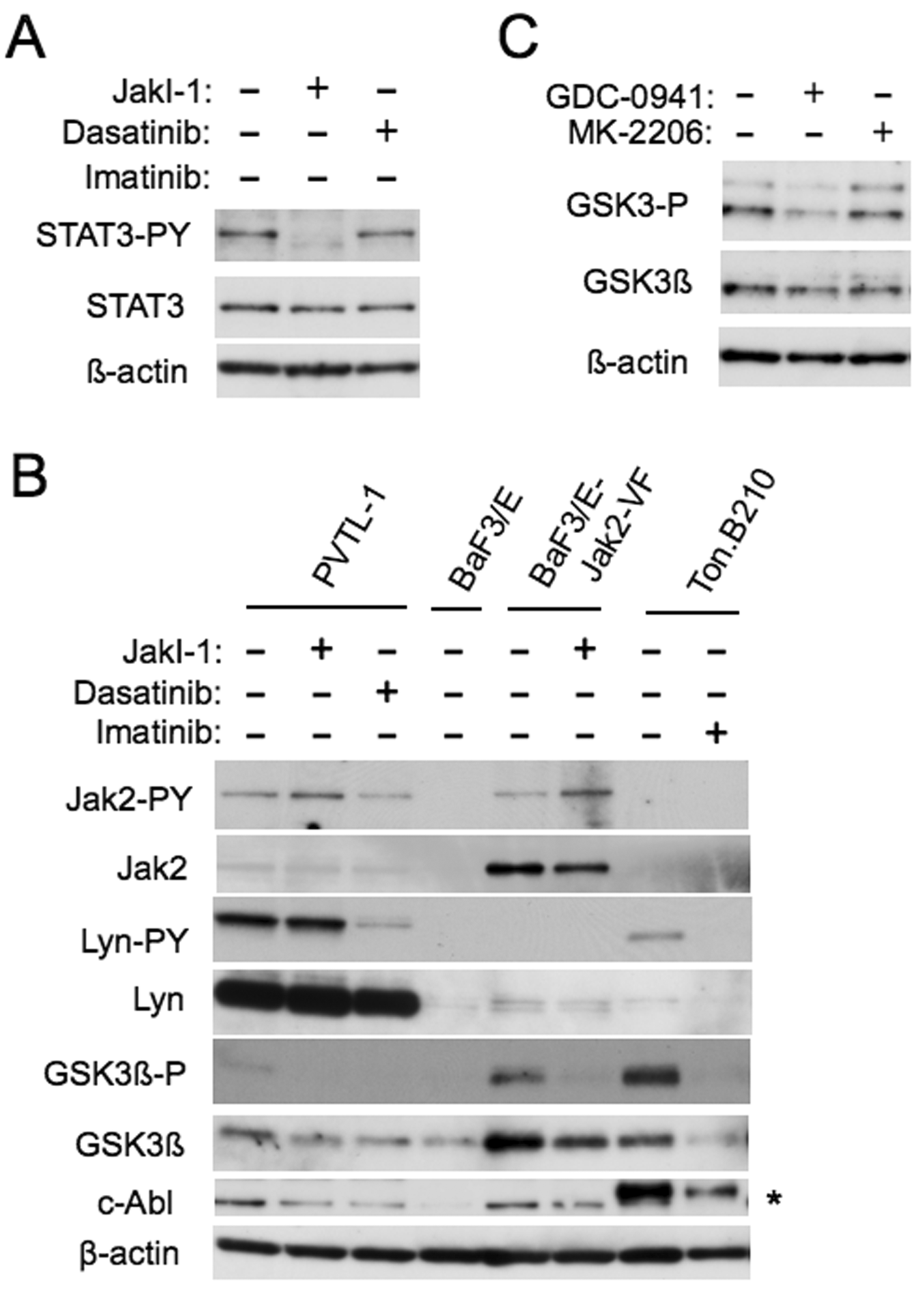

Supplement: Figure S1 — Effects of various inhibitors on intracellular signaling events in PVTL-1 and BaF3 cells expressing Jak2-V617F or BCR/ABL (A) PVTL-1 cells were left untreated as control or treated with 1 µM JakI-1or 50 nM dasatinib, as indicated, for 6 h and lysed. Total cell lysates were subjected to Western blot analyses with antibodies against indicated proteins. STAT3-PY: phospho-Y705-STAT3. (B) PVTL-1 cells treated as described above, BaF3/E cells starved from Epo for 6 h, BaF3/E/Jak2-VF cells treated with or without 2 µM JakI-1 for 1 h, and BCR/ABL-expressing Ton.B210 cells teated with or wihout 3 µM imatinib for 6 h, as indicated, were subjected to Western blot analyses with antibodies against indicated proteins. Abbreviations used are: Jak2-PY, phospho-Y1007/1008-Jak2; Lyn-PY, phospho-Y396-Lyn; GSK3ß-P, phospho-S9-GSK3ß. An asterisk indicates the position of BCR/ABL. (C) PVTL-1 cells were left untreated as control or treated with 5 µM GDC-0941 or 5 µM MK-2206, as indicated, for 6 h and subjected to Western blot analyses. GSK3-P: phospho-S21/9-GSK3α/ß. (TIF) [file pone.0084746.s001.tif]
